# The Mouse Papillomavirus Epigenetic Signature Is Characterised by DNA Hypermethylation after Lesion Regression

**DOI:** 10.3390/v13102045

**Published:** 2021-10-11

**Authors:** Allison M. Tschirley, Peter A. Stockwell, Euan J. Rodger, Oliver Eltherington, Ian M. Morison, Neil Christensen, Aniruddha Chatterjee, Merilyn Hibma

**Affiliations:** 1 Department of Pathology, Dunedin School of Medicine, University of Otago, Dunedin 9054, New Zealand; Allison.tschirley@otago.ac.nz (A.M.T.); peter.stockwell@otago.ac.nz (P.A.S.); euan.rodger@otago.ac.nz (E.J.R.); oliver.eltherington@otago.ac.nz (O.E.); ian.morison@otago.ac.nz (I.M.M.); aniruddha.chatterjee@otago.ac.nz (A.C.); 2 Department of Pathology, Pennsylvania State University, State College, PA 16802, USA; waipu6514@gmail.com

**Keywords:** DNA methylation, papillomavirus, epigenetics, hypermethylation, cutaneous squamous cell carcinoma

## Abstract

Papillomaviruses (PVs) are double-stranded DNA tumour viruses that can infect cutaneous and mucosal epidermis. Human papillomavirus (HPV) types have been linked to the causality of cutaneous squamous cell carcinoma (cSCC); however, HPV DNA is not always detected in the resultant tumour. DNA methylation is an epigenetic change that can contribute to carcinogenesis. We hypothesise that the DNA methylation pattern in cells is altered following PV infection. We tested if DNA methylation was altered by PV infection in the mouse papillomavirus (MmuPV1) model. Immunosuppressed mice were infected with MmuPV1 on cutaneous tail skin. Immunosuppression was withdrawn for some mice, causing lesions to spontaneously regress. Reduced representation bisulphite sequencing was carried out on DNA from the actively infected lesions, visibly regressed lesions, and mock-infected control mice. DNA methylation libraries were generated and analysed for differentially methylated regions throughout the genome. The presence of MmuPV1 sequences was also assessed. We identified 834 predominantly differentially hypermethylated fragments in regressed lesions, and no methylation differences in actively infected lesions. The promoter regions of genes associated with tumorigenicity, including the tumour suppressor protein DAPK1 and mismatch repair proteins MSH6 and PAPD7, were hypermethylated. Viral DNA was detected in active lesions and in some lesions that had regressed. This is the first description of the genome-wide DNA methylation landscape for active and regressed MmuPV1 lesions. We propose that the DNA hypermethylation in the regressed lesions that we report here may increase the susceptibility of cells to ultraviolet-induced cSCC.

## 1. Introduction

Human papillomaviruses (HPV) are a heterogenous, epitheliotropic, and ubiquitous group of non-enveloped viruses that range from harmless to cancer-causing. High-risk mucosal HPVs such as type 16 and 18 cause almost 100% of cervical cancers, as well as at least 30% of head and neck cancers [1]. Some HPV types infect cutaneous sites, causing hyperproliferation of skin cells that results in visible warts (papillomata), whereas infection with β-HPV types can occur in the absence of any visible lesion [2]. β-HPV types have been linked to the causality of cutaneous squamous cell carcinoma (cSCC), especially in the context of immune suppression [3,4]. However, this proposition is confounded by the observation that, unlike cervical cancer, β-HPV DNA is not always detected in the resultant tumour [5,6,7,8,9]. The lack of a need for the retention of viral DNA suggests that changes that support tumorigenicity occur in these cells following resolution of infection.

DNA methylation is an epigenetic change that can permanently alter expression of genes. It has been linked to many cancers, including cSCC [10,11,12], and some of the most frequently hypermethylated genes in cSCC include *CADM1*, *CDH1*, and *DAPK1* [10]. Several DNA tumour viruses, such as Epstein–Barr virus, Hepatitis B virus, and HPV dysregulate host gene DNA methylation [13]. Additionally, studies have described regulation of immune-related and tumour suppressor host genes by viral methylation following expression of selected HPV proteins in cells or have reported methylation patterns in cancer cells containing high-risk HPV E6 and E7 [14]. However, the extent of methylation of host genes that occurs during infection and after resolution of lesions is currently unknown.

Mouse papillomavirus (MmuPV1) is a pi papillomavirus that was first identified as a cutaneous infection in immune suppressed mice [15,16]. The MmuPV1 genome encodes seven open reading frames (ORFs) found in many HPV types (E1, E2, E4, E6, E7, L1, and L2). As with other papillomaviruses, each of the MmuPV1 ORFs is differentially expressed in the viral life cycle [16]. MmuPV1 does not contain an E5 ORF, similar to the cutaneous β HPV types [17]. Importantly, MmuPV1 encodes genes that have oncogenic potential. MmuPV1 E6 and E7 viral proteins increase proliferation of cells and tumorigenicity [18], and mice infected with MmuPV1 following ultraviolet (UV) irradiation can develop lesions that progress to squamous cell carcinoma [19,20]. Interestingly, although viral DNA was abundant in the initial warts in those lesions, very few cells contained amplified viral DNA once lesions had progressed to cSCC. MmuPV1 has recently been reported to frequently integrate into the mouse genome [21].

We hypothesise that viral infection alters methylation of the host DNA, with the premise that this may increase the tumorigenic potential of those cells. We tested this hypothesis in MmuPV1 infected cutaneous skin in immunosuppressed BALB/c mice, and methylation changes that occur during active lesion growth and following regression of lesions were measured. Interestingly, we found a high level of host DNA hypermethylation only after resolution of the visible lesion, and not in active lesions following MmuPV1 infection, compared to mock-infected control skin. We propose that the hypermethylation of host DNA we observe here contributes to an increased susceptibility to tumorigenesis following resolution of papillomavirus lesions.

## 2. Materials and Methods

### 2.1. Animals

Female specific pathogen-free (SPF) BALB/c mice, aged 8–12 weeks, were obtained from the Hercus Taieri Research Unit at the University of Otago and housed in a SPF environment. A total of 63 mice were initially in the study. This strain of mouse is considered moderately susceptible to MmuPV1 and only develop lesions when immune-suppressed.

### 2.2. Immune Suppression and Group Descriptions

For systemic immune suppression, 100 mg/mL Cyclosporin (CsA; Novartis Neoral^®^, Basel, Switzerland) diluted in phosphate buffered saline (PBS) was administered to 30 control and 30 infected mice by gavage five times per week (75 mg/kg, 11.25 mg/mL), starting one week prior to MmuPV1 infection. Three mice were non-CsA treated control mice. Groups consisted of infected and actively increasing lesion mice (A) and MmuPV1 infected then regressed (R). Their mock-infected or mock-infected then regressed paired samples were denoted (C)A and (C)R, respectively. A comparison between CsA treated and untreated mice (C) was performed to assess any methylation differences due to immune suppression. Methylation effects of scarification were also controlled for by comparing the non-scarified region of mock-infected (C)A and the CsA treated groups. Commencing at day −3, Baytril (Bayer Comporation, Leverkusen, Germany) was added to drinking water for all immune suppressed mice to reduce the risk of bacterial infection.

### 2.3. Ethics

All animal experiments were approved by the Animal Ethics Committee at the University of Otago (ethical approval 12 Nov 2014; AEC56/14), were performed in accordance with the New Zealand Animal Welfare Act (1999), and adhered to the National Animal Ethics Committee (Ministry of Primary Industries, New Zealand) guidelines. 

### 2.4. MmuPV1 Virus and Infection Protocol

**Figure 1:**
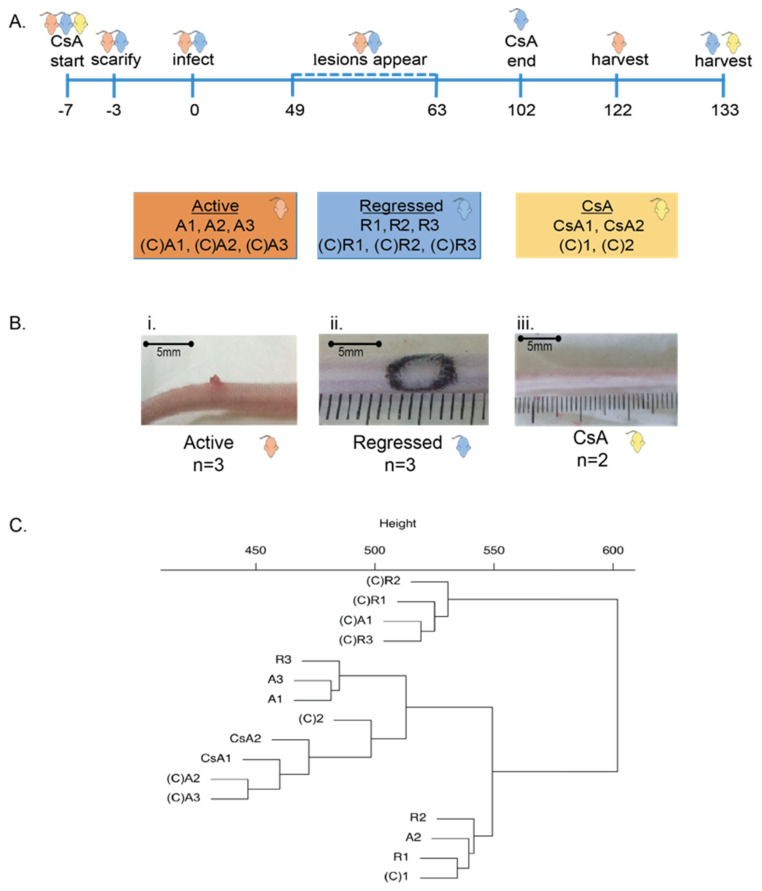
Experimental setup for the mouse model and preliminary methylation analysis. (**A**) Timeline of infection model. Colour-coded mouse images represent the three groups as shown in the boxes below. Boxes show sample names of each group and their associated control samples. (**B**) Representative images of the experimental groups show mouse tails at sample location; (i) Active viral infection, (ii) Regressed lesion (original lesion location delineated in black), (iii) CsA treated, uninfected. (**C**) Hierarchical clustering dendrogram showing the difference in total methylation of all high-coverage autosomal fragments for each of the 16 samples. A and R indicate actively MmuPV1 infected and MmuPV1 infected then regressed, respectively. (C)A and (C)R indicate mock infected and mock infected then regressed, respectively. CsA and (C) indicate CsA treated and untreated, respectively. Except for one sample, the largest difference in methylation is between four mice that were mock infected and three mice that were infected with MmuPV1.

For the initial infection of mice, mouse papillomavirus was previously isolated from lesions on the tails of Foxn1^nu^/Foxn1^nu^ mice and diluted 1:10 in PBS, as previously described, was used [22]. Briefly, lesions were scraped from the tail and homogenised, and the supernatant was collected. Viral supernatant was then diluted with PBS and passed through a sterile syringe filter before storage at –20 °C. For infection of the mice, tail skin was scarified using a hand-held rotary tool with a felt wheel attachment on day −3 [23]. Animals were anaesthetised with Isoflurane (Bayer Corporation, Leverkusen, Germany), a 1.5 cm long mark was placed at the base of the tail using a felt tip pen, and the rotary tool was moved 10 times along the mark at a speed of approximately 15,000 rpm to remove the upper epidermal layer. Marcain (Pfizer, New York City, NY) was applied dropwise over the wound area for pain relief. At day 0, mice were placed in restrainers, and the scarified region was scored lightly with a needle tip. Inoculation was performed by placing 20 µL of prepared virus onto the scored region of the scarified area (Figure 1A). Mock-infected mice received PBS. At day 49, animals who had yet to show signs of a lesion were re-infected with 20 µL of native tail virus isolated from lesions on the tails of BALB/c mice, diluted 1:5 in PBS and sterile filtered. At day 102, CsA was discontinued for the regressed group, to allow the lesions to heal naturally. 

### 2.5. Lesion Monitoring and Harvesting

Mice were monitored weekly for signs of lesions. Measurements of lesions were taken either immediately prior to harvesting or, for lesions in the regressed group, immediately prior to withdrawing CsA. A lesion was “actively increasing” if its largest volume measurement was its last one. A lesion was considered “partially regressed” if it had reached its greatest volume and was decreasing in volume. A lesion that was “fully regressed” had appeared during the study but was no longer overtly present. Beginning at day 102 for the regressed group, the lesions were outlined with marker pen for identification of tissue to be harvested once lesions regressed. The actively increasing lesion group mice and their mock-infected controls were culled at day 122 post-infection. Tail skin was removed, and epidermal dissociation of the epithelium was performed following the protocol of Lichti et al. [24]. DNA was extracted from epidermal tissue (approximately 3–5 mm^2^ per lesion) using a DNAeasy blood and tissue kit (Qiagen; Hilden, Germany). Regressed and CsA group mice and their controls were culled at day 133 post-infection once lesions were no longer palpable (31 days post withdrawal of CsA for regressed group). Lesion tissue was harvested as described for the actively infected group above.

### 2.6. Reduced Representation Bisulphite Sequencing Library Preparation

Reduced representation bisulphite sequencing (RRBS) was performed on the 16 samples of genomic DNA extracted from tail epidermal tissue as previously described [25,26,27]. Briefly, DNA was digested into fragments overnight using the MspI restriction endonuclease. The DNA was then purified following QIAquick kit (Qiagen, Hilden, Germany) instructions. Subsequently, DNA was end-repaired by incubation for 30 min with Nano End Repair Mix 2 (Illumina; San Diego, CA, USA) and again purified using the MinElute PCR purification kit (Qiagen; Hilden, Germany). Adenylation of 3′ ends was followed by ligation of sequencing adaptors, with a specific index to identify each sample. The fragments were size selected (150–325 bp) by excising the appropriate bands from 3% Nusieve agarose gels. These fragments were then bisulphite converted using EZ DNA methylation kit (Zymo Research; Irvine, CA, USA) prior to a PCR amplification step. The quality of the 16 libraries was confirmed using bioanalyser traces before being sequenced on an Illumina HiSeq2500 machine (Table 1).

### 2.7. DNA Methylation Analysis

Differential methylation analysis was performed using the previously reported differential methylation analysis package (DMAP) [28,29,30]. Briefly, we used a fragment-based approach, using the MspI fragments that had been digested in the RRBS library preparation as the unit of analysis for identifying variable methylation. Only fragments with at least two CpG sites and covered by at least ten sequenced reads per CpG were included in the data (hereon called high coverage fragments). High coverage fragments were mapped to the mouse genome and the closest protein coding genes were annotated. 

### 2.8. Group Comparisons

Five different group comparisons were performed on the data produced by DMAP; regressed vs. control, active vs. control, active vs. regressed, CsA vs. no CsA, scarified vs. non-scarified (Figure 1B). Active and regressed tissues were the experimental groups. Non-scarified vs. scarified was a methylation comparison only and compared animal samples from the CsA and actively increasing lesion control groups. These control tissues were tested to determine whether scarification impacted on methylation. CsA vs. no CsA tissues was assessed to determine whether CsA administration impacted methylation.

### 2.9. Analysis of Viral Reads and Pathway Analysis

Methylation data were aligned to the MmuPV1 genome using bowtie in the Bismark Bisulphite Read Mapper [31]. Sequences that mapped to the MmuPV1 genome were also aligned to the *Mus musculus* genome to ascertain whether mapping was unique to the viral genome. The mapper BSMAP(z) was then used to verify the alignments shown by Bismark. This mapping software confirmed that the reads were not indicating artefactual behaviour of Bismark alone. The Integrative Genomics Viewer (IGV) was used to visualise reads within the MmuPV1 genome [32]. Lanes were auto-scaled to show proportionate representation between the samples.

### 2.10. Statistical Analyses

All data are presented as the mean ± standard error of the mean and all statistical analyses of methylation data were done in GraphPad Prism v7^®^ (Graphpad Software, San Diego, CA, USA). For methylation analysis, an ANOVA test was applied to the fragments of the experimental and control groups, and regions showing methylation differences with a fold change of ≥ 1.5 and significant *p*-values were identified. Mann–Whitney U was used to test for differences between groups. The Benjamini-Hochberg false-discovery rate (FDR) method under the multiple t-tests function was applied to increase the power of the data and identify significant *p* values with an FDR of 5%, as indicated by the volcano plots. Unsupervised hierarchical clustering was performed in the R environment using the Euclidean distance metric of all analysed high coverage autosomal fragments (*n* = 126,681) from the 16 RRBS methylomes. This shows samples with bigger differences in total amount of methylation farther away from each other in the hierarchy. Pathway analysis was performed on a gene list containing the 221 genes differentially expressed in the core promoter region using the online platform Enrichr [33]. The enrichment *p* values calculated by Enrichr are from a modified Fisher’s exact test, which is a proportion test that assumes a binomial distribution and independence for probability of any gene belonging to any set.

## 3. Results

### 3.1. Immune-Suppressed BALB/c Mice Develop and Maintain Active MmuPV1 Infection

Thirty mice that were CsA treated were infected with MmuPV1 and were monitored for the development of visible lesions and another thirty CsA treated control mice were mock-infected with PBS (Figure 1A). Infected mice that had not developed visible lesions were re-infected at day 49. Out of 30 total mice, six developed lesions visible to the naked eye between days 49 and 63 post-infection, with a mean lesion length of 2.4 ± 0.3 mm (Figure 1B). The remainder of the infected mice did not develop visible lesions or developed lesions that spontaneously regressed while the animal was still immune-suppressed and were therefore excluded from the study. The final groups consisted of, firstly, mice (*n* = 3) that were harvested at day 122 with “actively increasing” lesions (A1, A2, A3) and three matched mock-infected controls ((C)A1, (C)A2, (C)A3), and secondly, mice that produced active lesions but were harvested at day 133 with “regressed” lesions (R1, R2, R3) following the withdrawal of CsA at day 102, and three matched CsA treated, mock-infected controls ((C)R1, (C)R2, (C)R3). All mice in the regressed group had healed their lesions (assessed by visual and tactile tests) by 31 days post CsA withdrawal. Lesion length measurements taken immediately prior to CsA withdrawal for the regressed group were 2 mm, 1.5 mm, and 3 mm for R1, R2, and R3, respectively. Lesion length measurements for the active group were 3 mm, 2mm, and 3 mm for A1, A2, and A3, respectively. Groups of two CsA treated mice (CsA1, CsA2) and two controls ((C)1, (C)2) were included in the study to test whether any changes in DNA methylation occurred as a result of CsA treatment alone. 

RRBS was carried out on all 16 tissue samples. On average, we generated 9.3 × 10^6^ ± 0.5 × 10^6^ uniquely aligned reads for each of the samples tested (Table 1). The mean mapping efficiency was 47 ± 1.3%, which is consistent with the expected level of efficiency for a bisulphite converted genome. 

To assess the relationship of global RRBS methylation between the samples that were tested, we performed hierarchical clustering of all analysed high coverage autosomal fragments from the 16 RRBS methylomes in this study (Figure 1C). We found that all the infected samples (A1-3, R1-3) grouped into two clusters, irrespective of whether they were actively increasing lesions or if the lesion had regressed (Figure 1C). Samples from uninfected mice ((C)A1-3, (C)R1-3, CsA1-2, (C)1-2) generally grouped into two other clusters, separated from the infected mice.

An analysis of differentially methylated fragments (DMFs) was carried out between test and control groups. The criteria for the inclusion of a DMF was a fold change difference of ≥ 1.5, a significant *p*-value (*p* < 0.05), and a false discovery rate (FDR) of 5% or less using the stringent Benjamini–Hochberg multiple test correction. We were surprised to see that no significantly DMFs were identified for actively increasing lesions (A1-3 c.f. (C)A1-3; Table 1). As might be predicted, treatment of mice with CsA (CsA1-2 c.f. (C)1-2) or the scarification of tissue alone ((C)A1-3 c.f. CsA1-2) had minimal effects on differential methylation. In contrast, 834 DMFs were identified when we compared tissues from lesions that had regressed compared to matched control tissues.

### 3.2. Genome-Scale DNA Methylation Identifies Extensive Hypermethylation in Regressed Lesions 

**Figure 2:**
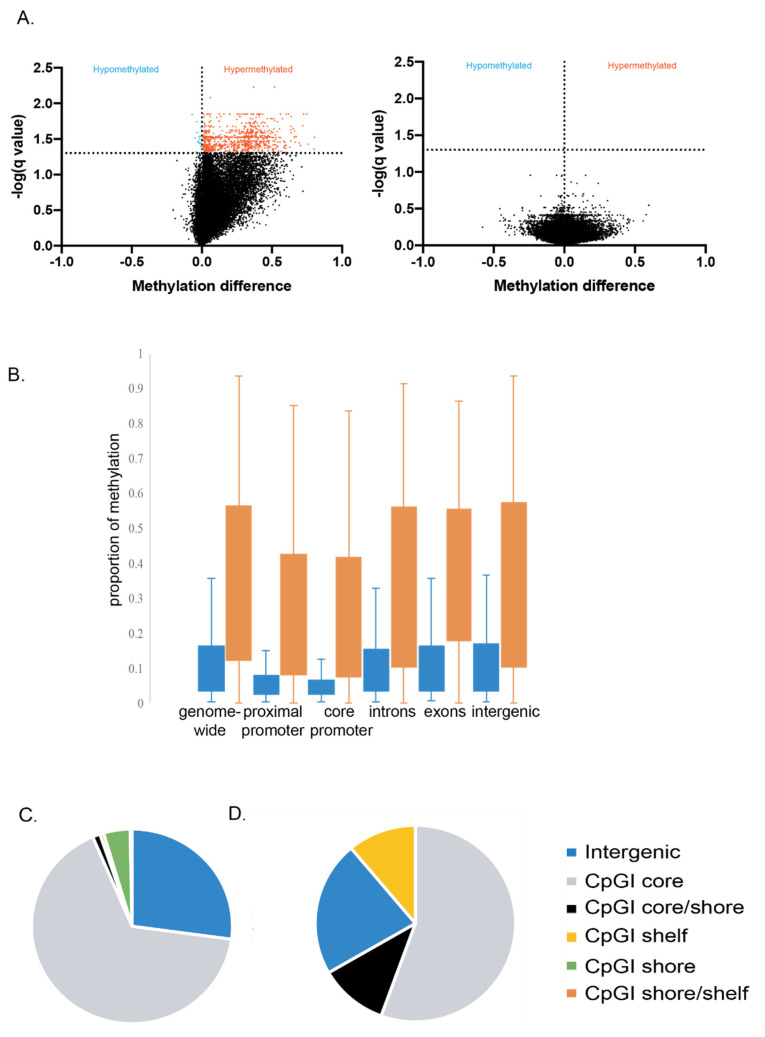
Global methylation description. (**A**) Volcano plots of differentially methylated fragments (DMFs) in comparison of MmuPV1 infected then regressed to mock infected then regressed control tissue (left) and actively infected to mock infected control tissue (right). Horizontal dotted line represents Benjamini-Hochberg false-discovery rate of 5% to control for multiple t-tests. Vertical dotted line represents a mean difference of 0 in the proportion of methylation between groups; (**B**) Proportion of methylated DMFs by genomic region for regressed control tissues (blue) and regressed (orange). Data are represented in quartiles with whiskers indicating outliers outside of upper and lower quartiles. (**C**) Within CpG islands, differentially hypermethylated regions (*n* = 328) and (**D**) hypomethylated regions (*n* = 9) were located predominantly in the core.

We carried out further analysis to determine if the DMFs were hyper or hypomethylated. The striking finding from this analysis was that 98% of the DMFs identified in the regressed group were significantly hypermethylated (Mann–Whitney U, *p* < 0.0001) (Figure 2A), whereas none of the methylated fragments in the actively increasing lesion group were significantly hyper or hypomethylated. 

To further probe the DMFs in the regressed MmuPV1 lesions relative to their location in the genome, we generated DNA methylation maps for the specific genomic regions. We found that methylation was increased overall in the DMFs from all genomic regions that we assessed in regressed skin when compared with controls (Figure 2B). Significantly increased methylation was detected in DMFs genome-wide (Mann–Whitney U, *p* < 0.0001), proximal to gene promoters (defined as ±1 kb of transcription start site) (Mann–Whitney U, *p* < 0.0001), in the core promoter region (defined as ±500 bp of transcription start site) (Mann–Whitney U, *p* < 0.0001), in gene introns (Mann–Whitney U, *p* < 0.0001) and gene exons (Mann–Whitney U, *p* < 0.0001), and in intergenic regions (Mann–Whitney U, *p* < 0.0001). 

To further investigate the methylation landscape, we performed an analysis of the wider CpG island topography (10 kb upstream to 1 kb downstream of transcription start sites). We found that most of the hypermethylation was in the CpG island cores, being defined as stretches of DNA 500–1500 bp long with a CG:GC ratio of more than 0.6 [34]. Of 328 hypermethylated fragments detected within the region of interest (Figure 2C), 66% were found in the core, 4% were in the CpG island shore (up to 2 kb upstream and 2 kb downstream of the CpG island core), 1% were in the interface between core and shore (termed core-shore), and <1% were in the shelf (up to 2 kb upstream and 2 kb downstream of the shore). Additionally, 27% of the regions were outside of these CpG island features. Of the nine hypomethylated fragments that were detected (Figure 2D), five were in the CpG island core.

### 3.3. Genes in the Core Promoter Region Are Skewed Extensively towards Hypermethylation in Regressed Lesions

**Figure 3:**
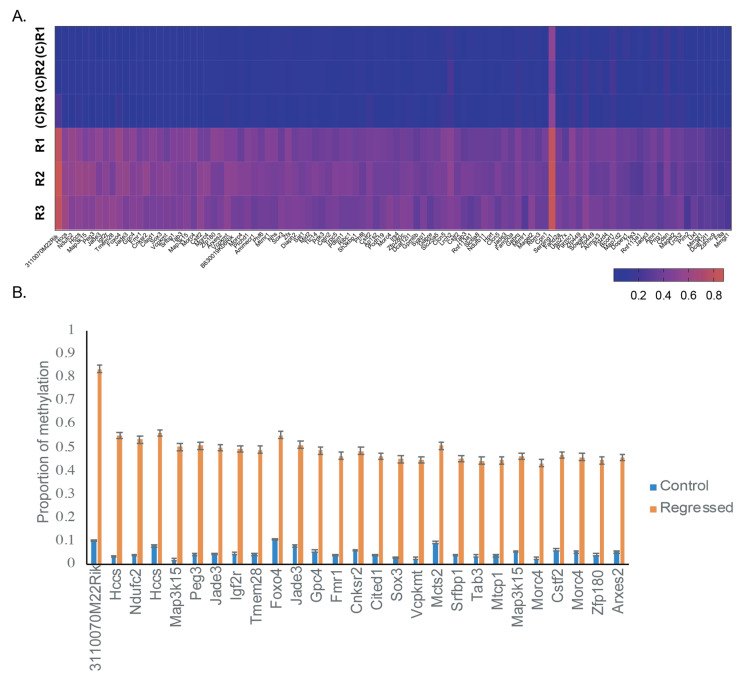
Extensive hypermethylation identified in regressed skin. (**A**) Individual DMFs in the core promoter region of genes with >20% methylation difference between regressed and control groups by individual sample. Five genes with insufficient data to calculate methylation in at least one sample have been removed from the heatmap (**B**) Individual DMFs in the core promoter region of genes with >40% methylation difference between regressed and control groups. More than one DMF was identified for some genes. Error bars display standard error of the mean.

As promoter DNA methylation strongly influences expression from the corresponding gene, we specifically investigated differential methylation in the core promoter region. Of the 221 DMFs in the core promoter region, 214 were hypermethylated, and seven were hypomethylated. When comparing individual samples within the groups, all 100 genes with >20% methylation difference were hypermethylated in the regressed group (Figure 3A). This was also the case at > 40% methylation difference (Figure 3B), where all 27 genes were hypermethylated in regressed tissue compared to control tissue.

### 3.4. Hypermethylated Genes in Regressed Lesions Are Enriched for Cellular Senescence and Cancer Related Pathways and for CTCF and RNA Polymerase II Binding

**Figure 4:**
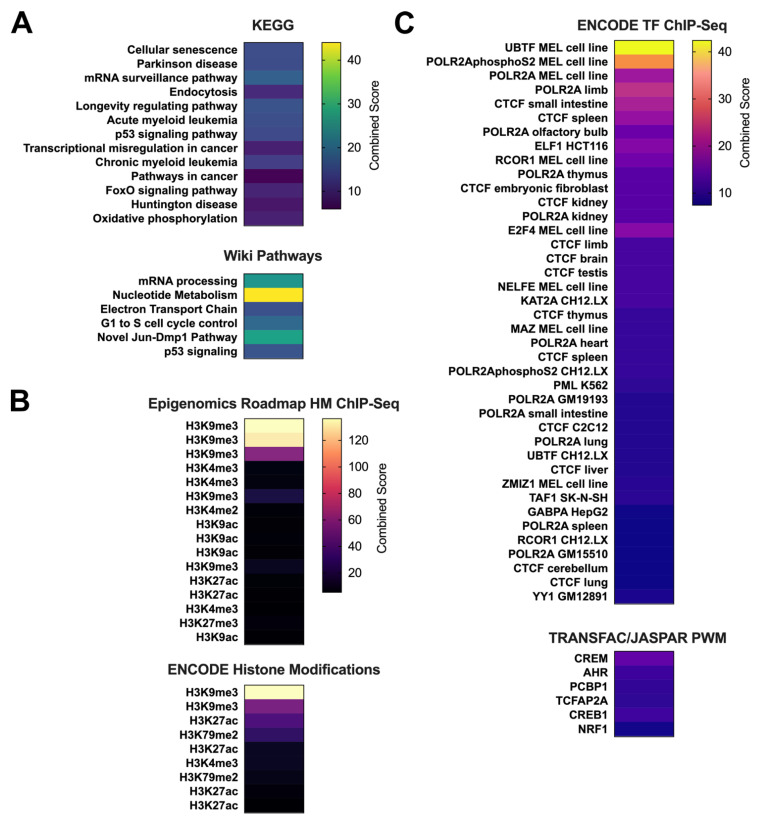
Pathway and transcriptional function analysis of differentially methylated fragments (DMFs). Enrichr analysis with default parameters was used to identify pathways, histone modifications and transcription factor targets associated with DMFs in core gene promoter regions. Individual heatmaps show: (**A**) enriched KEGG and Wiki Pathways (*p* < 0.05); (**B**) enriched histone modifications from Epigenomics Roadmap and ENCODE datasets (*p* < 0.01); (**C**) Transcription factor targets from ENCODE ChIP-Seq and TRANSFAC/JASPAR positional weight matrices (*p* < 0.01).

To document the function of the genes that were hypermethylated in the core promoter regions of regressed skin, we performed functional enrichment analysis using two different sources (KEGG and Wiki Pathways for Mouse). We found genes involved in cellular senescence/cell cycle, mRNA processing, and p53 signalling were significantly enriched (*p* < 0.05) in both the analyses (Figure 4A). Disease associated pathways, particularly cancer pathways, were also enriched in our analysis. To place our findings in a broader epigenomic context, we utilised histone modification ChIP-Seq data (consisting of active and repressive histone marks) from the ENCODE project as well as from the Epigenomics roadmap (Figure 4B). The genes harbouring hypermethylation in the core promoters of regressed skin were predominantly enriched for the repressive chromatin mark H3K9me3. We also found significant enrichment for several active histone marks, especially H3K27ac and H3K9ac (Figure 4B). Overlap analysis of ENCODE transcription factor ChIP-seq data identified enrichment of several transcription factors for the hypermethylated genes (Figure 4C). The most frequently identified transcription factors were *CTCF* (which encodes a zinc-finger binding protein that is a transcriptional and chromatin regulator) and *POLR2A* (encoding the RNA Polymerase II Subunit). 

### 3.5. MmuPV1 Viral Reads Are Highly Concentrated in the E4 Portion of the Genome

**Figure 5:**
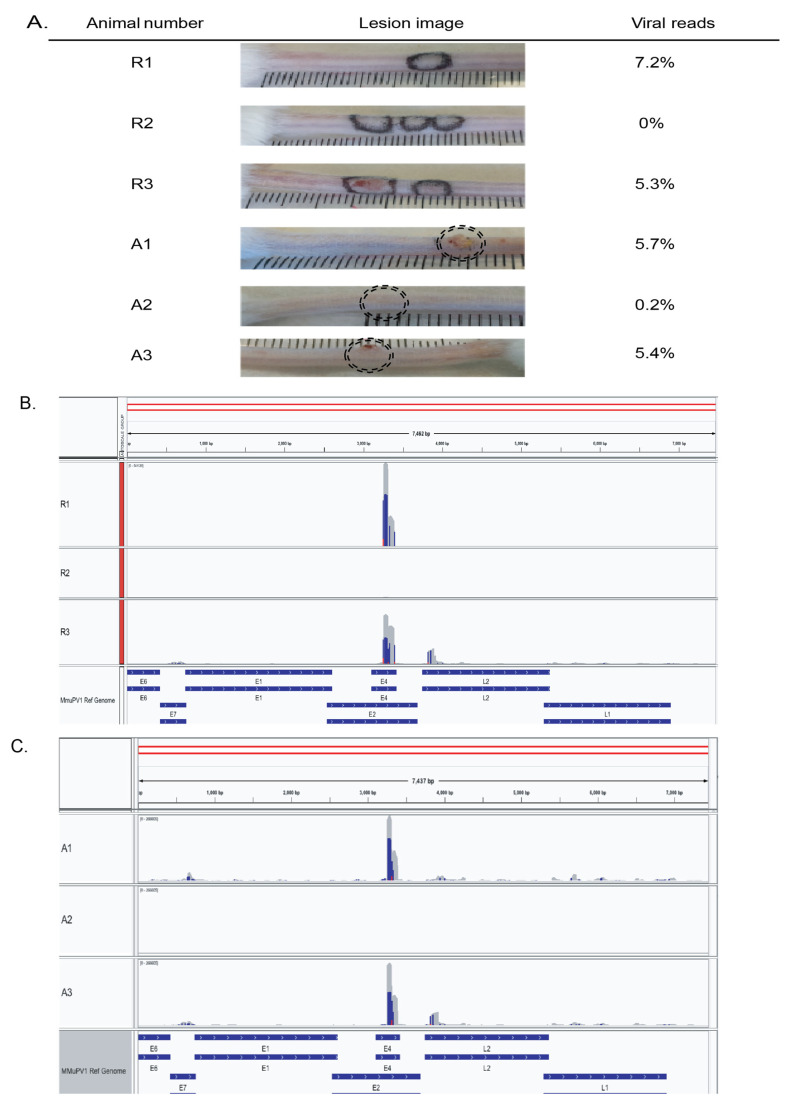
MmuPV1 viral reads within individual samples. (**A**) Images of mouse tails at time of harvest. Marker pen was used to delineate the area of the lesions at the height of infection. (**B**,**C**) Coverage and distribution of viral reads visualised using IGV software. Coverage depth of reads set to group autoscale for (**B**) regressed (0–266,605) and (**C**) active groups (0–541,120).

We probed the data for MmuPV1 viral reads to determine whether viral DNA remained after lesion regression (Figure 5A). Traces of the viral genome were present in all actively increasing tissue (5.7%, 0.2% and 5.4% of reads were viral in origin for samples A1 to A3, respectively). Interestingly, viral reads were also detected in two of three regressed tissues (7.2%, 0.0% and 5.3% for samples R1 to R3, respectively). The small number of background reads mapping to the MmuPV1 genome in uninfected groups (Table 2) most likely occurred through the misalignment of bisulphite reads, as previously reported [35]. The most intriguing finding of viral read analysis was the high proportion of viral reads within the E4 region of the MmuPV1 genome relative to other regions of the genome (Figure 5B,C). In two of the actively increasing lesion samples (A1 and A3), more than half of all reads aligned to this region. In one of the regressed lesions, sample R1, 74% of the viral reads mapped to E4. The L2 region also had high clustering, contributing up to 25% of all viral reads in the actively increasing lesion samples. 

## 4. Discussion

Here, we show the first reported analysis of global and gene-specific DNA methylation during MmuPV1 infection and following regression of the lesion. Interestingly, differential methylation was only detected following lesion regression, and not during actively increasing lesions. This differential methylation was highly skewed towards hypermethylation.

Hypermethylation has been observed in the pre-cursor lesions of cervical cancer, with the increasing methylation of *CCNA1* from low grade to high grade cervical lesions being suggested as a diagnostic marker for progression [36]. DNA methylation is variable during cervical carcinogenesis, with maximal variation in DNA methylation in “risk” CpG loci immediately prior to the onset of cervical cancer [37]. Additionally, HPV-associated head and neck cancer tumour growth was found to be suppressed by methylation inhibitors [38]. Thus, hypermethylation has been implicated in HPV-related cancers. 

In our analysis, *DAPK1* was hypermethylated in core promoters in regressed skin compared to control tissue. *DAPK1* is a tumour suppressor protein down-regulated in many cancer types [39]. Li and colleagues found that hypermethylation of *DAPK1* was associated with cSCC [10]. Although Li et al. found no correlation between hypermethylation of *DAPK1* and the presence of cutaneous HPV, *DAPK1* hypermethylation is implicated in other HPV associated human cancers, such as cervical cancer [40]. The hypermethylation of *DAPK1* that we observed here suggests that this gene may be “switched off” in MmuPV1-regressed lesions; however, changes in protein levels as a result of hypermethylation needs to be confirmed for this and the other hypermethylated genes that we have identified.

The identification of hypermethylation of *POLR2A* shown here is consistent with the observation that one of the enriched pathways that we identified was mRNA processing, suggesting a role of hypermethylation in RNA processing in regressed skin. We also identified hypermethylation of the *CTCF* promoter. Variable *CTCF* binding has been strongly linked with differential DNA methylation in multiple human cell types, and it has been shown that *CTCF* binding patterns are markedly different in normal versus immortal human cells. In immortal cells, disruption of *CTCF* binding was strongly associated with hypermethylation [41]. Our analysis provides evidence for hypermethylation of *CTCF* which may lead to disruption of *CTCF* binding in regressed skin compared to normal skin controls. 

Although our study did not investigate the effects of UV on MmuPV1 infection or lesion progression, UV is known to be a mutagen and an immunosuppressant and important in the causality of cSCC [42]. Uberoi et al. [20] found that immune-competent FVB mice infected with MmuPV1 and exposed to UV radiation were more likely to develop lesions, and that some of those lesions progressed to cSCC. Around 58% of FVB mice that were exposed to UVB either 24 h prior to, or 24h post-infection developed papillomas by 12 weeks; however, this was strain specific and could not be replicated in BALB/c mice. Some of the lesions (between 37% and 58%) harvested at 6 months showed some evidence of malignant progression. They concluded that UVB increases susceptibility to MmuPV1 disease by inducing immunosuppression, but their data also support a relationship between UVB, MmuPV1, and the development of cSCC. 

Defective DNA mismatch repair following UV exposure can contribute to cSCC. We found that the DNA mismatch repair genes *MSH6* and *PAPD7* were hypermethylated within the core promoter region of regressed skin (5% and 6.5% methylation difference, respectively). *MSH6* is important for UVB-induced apoptosis [43,44]. Its hypermethylation indicates that the damage response no longer occurs. Key genes such as *MSH6* and *PAPD7*, whose promoters are hypermethylated in regressed MmuPV1 lesions, may render the skin more sensitive to the damaging effects of UV.

It is interesting to note that the methylation changes that we observe here only occurred after regression of the visible lesion, not during the active infection. Although it seems most likely that these DNA methylation changes occur in keratinocytes and as a result of the previous infection, there are other possible explanations. For example, epigenetic changes that include DNA methylation are associated with the healing response [45]. CsA withdrawal did not result in was not the source of the methylation changes we observed here, as this was controlled by the CsA also being withdrawn from the matched control mice. Although the predominant source of the DNA in this study is keratinocytes, some infiltration into the epidermis of immune cells that are specifically associated with immune-mediated regression of the lesion may contribute to changes in DNA hypermethylation. Mitra et al. (2020) reported distinct immune methylation signatures in metastatic melanoma that related to patient survival, and it is feasible that the DNA hypermethylation that we measured in this study may reflect changes in infiltrated immune cells [46].

A limitation of this study is that the experimental group sizes were smaller than planned (*n* = 3), because the number of mice that developed visible papillomas in our study was about a third of what had been reported by others. It has previously been reported that BALB/c mice demonstrate intermediate susceptibility to MmuPV1 infection when immunosuppressed with CsA [47]. In that study, five out of eight (63%) BALB/c mice produced MmuPV1 lesions following infection. In our study, the proportion of mice that developed lesions was much lower (20 %), and some mice required a second dose of virus for a visible lesion to appear. The differences between these studies may be attributable to differences in the amount of virus administered or in the administration of the CsA treatment (oral gavage c.f. subcutaneous injection) between the two studies. Regarding the group sizes, we acknowledge that other studies reporting methylation in mouse skin typically use slightly larger group sizes (*n* = 5) [48,49]. In MmuPV1 infectivity studies, Xue et al. (2017) used *n* = 3 for their analyses, but with three sites per mouse, and Wei et al. (2020) used group sizes of 3–8 mice for tongue infections using MmuPV1 [50]. We note that hypermethylation in the regressed mice was pronounced and consistent in all three animals in our study and was not detected in any of the actively infected mice, giving us confidence in the validity of these data. Furthermore, statistical rigor was applied in the methylation analysis, with an FDR of 5% on Benjamini–Hochberg-corrected data. However, we also note that in HPV16-E5 transgenic mouse exophytic papillomas developed following dimethylbenz[a]anthracene/12-O-tetradecanoyl/phorbol-13-acetate (DMBA/TPA) treatment but that the carcinomas that developed were flattened out endophytic lesions. Although these models differ in several regards, it is possible that the regressing lesions were flattened out endophytic cSCC lesions [51]. This should be confirmed in future studies.

Our analysis of viral reads found evidence of viral DNA in regressed tissues with no remaining visible lesion. Viral gene expression was detected by Xue et al. [52] well before lesions were visible. These investigators observed that the viral copy number did not directly correlate with lesion size. This was similarly found in our analysis of viral reads and the relationship with lesion size, where one visible lesion in the actively increasing lesion group (sample A2) yielded only 0.2% viral reads, while skin with no visible lesion in the regressed group (sample R1) showed the highest level of viral DNA at 7.2%. A limitation of using RRBS data to look at viral reads is that we cannot visualise the whole genome, only fragments, and consequently, we cannot comment on copy numbers or integration of the viral genome.

The low viral reads of sample A2 could be partly explained by the size of the lesion, which was smallest of the three actively increasing lesions (height: 0 mm, length: 2 mm, width: 1 mm) and could contribute to its methylation profile clustering with two of the samples from the regressed lesion group (Figure 1C). Interestingly, R1, which had the highest level of viral reads, clustered with A2 (0.2%), R2 (0%), and (C)1 (did not receive virus). Except for (C)1, this shows that the largest difference in methylation is between four mice that were mock infected and three mice that were infected with MmuPV1, suggesting that infection status, not level of viral reads, is more important in methylation differences. Rabbit oral papillomavirus DNA and RNA has been found to persist for at least a year post lesion regression [53]. The high viral reads in two out of three regressed lesions suggests viral latency in MmuPV1 is possible, or that viral integration has occurred. The diversity in the quantity of viral DNA found could be explained by the stage of epithelial differentiation that the cells were in when collected, as productive papillomavirus infection is dependent on the differentiation of the host epithelial cell. A progression of this research could involve re-establishment of immunosuppression in regressed animals to see if the latent infection re-emerges at the site of the original lesion.

One of the striking findings within the analysis of viral reads was the high clustering of reads in the E4 region. E4 protein is found to be abundant in upper layers of the epithelium of productive lesions [54]. E4 expression is followed by the late structural proteins, explaining the concurrent, though considerably lower, expression of L2. This pattern was also found by Xue et al. [52], who looked solely at active MmuPV1 infections in the tail, muzzle, and ear of mice. 

There is considerable variability in the pattern of expression of different papillomavirus types, depending on the animal model and tissue tropism [55]. High levels of E4 transcripts in the upper layers of the epithelium, similarly to the DNA viral reads found here, follow the pattern found in low-grade squamous intraepithelial lesions of high-risk cervical HPV infections [56]. In an analysis of 92 different HPV genomes, the E4 region was found to have the highest proportion of CpG sites, sites where methylation predominantly occurs [57]. This is even though CpG sites tend to be under-represented in viruses, which may be a way to avoid methylation by host methyltransferases or CpG-mediated immune responses. 

In summary, our study demonstrates the notable hypermethylation and the retention of some viral DNA in previously MmuPV1-infected tissue following resolution of the visible lesion. We highlight several genes hypermethylated in the core promoter regions of regressed skin that we speculate could influence susceptibility of those cells to UV-induced cSCC.

## Figures and Tables

**Table 1:** Methylation alignment and efficiency by individual sample and number of differentially methylated fragments (DMFs) using Benjamini–Hochberg correction for each group comparison.

Comparison Groups	Individual	No. of Unique Alignments	Mapping Efficiency	No. of DMFs at 5% FDR
Regressed	R1	6,942,498	44.3%	834
	R2	7,861,430	48.6%	
	R3	7,240,969	47.0%	
Regressed Control	(C)R1	11,964,235	50.6%	
	(C)R2	12,197,807	49.8%	
	(C)R3	9,824,427	39.5%	
Actively increasing lesions	A1	7,449,228	49.6%	0
	A2	10,712,796	33.7%	
	A3	6,442,790	49.5%	
Active Control	(C)A1	13,217,138	38.3%	
	(C)A2	9,198,760	49.8%	
	(C)A3	10,374,183	51.9%	
No CsA control	(C)1	6,434,780	47.8%	0
	(C)2	6,853,519	49.1%	
CsA	CsA1	9,428,602	49.5%	
	CsA2	12,426,447	52.7%	
Non-scarified	CsA1, CsA2			0
Scarified	(C)A1, (C)A2, (C)A3			
Active	A1, A2, A3			0
Regressed	R1, R2, R3			

**Table 2:** Total viral reads and percent viral reads within E4 region in active, regressed and control tissues.

Group	Sample	Total Reads	Viral Reads	% Viral Reads	% in E4	Infection Status
Regressed lesions	R1	15,674,849	1,129,427	7.2	74	Regressed lesion
	R2	16,166,560	5871	0	0	Regressed lesion
	R3	15,392,780	819,114	5.3	59.9	Regressed lesion
Regressed Control	(C)R1	23,666,799	3713	0	0	Mock-infected
	(C)R2	24,496,535	2868	0	0	Mock-infected
	(C)R3	24,877,419	4143	0	0	Mock-infected
Actively increasing lesions	A1	15,020,374	849,677	5.7	52.1	Active lesion
	A2	31,835,719	48,521	0.2	11.4	Active lesion
	A3	13,025,548	708,976	5.4	56	Active lesion
Active Control	(C)A1	34,512,945	6246	0	0	Mock-infected
	(C)A2	18,479,427	2534	0	0	Mock-infected
	(C)A3	19,996,210	2475	0	0	Mock-infected
CsA	CsA1	19,044,226	1271	0	0	CsA/No infection
	CsA2	23,584,967	1829	0	0	CsA/No infection
No CsA Control	(C)1	13,470,444	1106	0	0	No CsA/Not inf.
	(C)2	13,946,497	1282	0	0	No CsA/Not inf.

## Data Availability

The RRBS data generated as part of this study were submitted to Gene Expression Omnibus (GEO) with the accession number GSE160868.
